# Brain Susceptibility Changes in a Patient with Natalizumab-Related Progressive Multifocal Leukoencephalopathy: A Longitudinal Quantitative Susceptibility Mapping and Relaxometry Study

**DOI:** 10.3389/fneur.2017.00294

**Published:** 2017-06-19

**Authors:** Giuseppe Pontillo, Sirio Cocozza, Roberta Lanzillo, Pasquale Borrelli, Anna De Rosa, Vincenzo Brescia Morra, Enrico Tedeschi, Giuseppe Palma

**Affiliations:** ^1^Department of Advanced Biomedical Sciences, University “Federico II”, Naples, Italy; ^2^Department of Neurosciences, Reproductive and Odontostomatological Sciences, University “Federico II”, Naples, Italy; ^3^IRCCS SDN, Naples, Italy; ^4^Institute of Biostructure and Bioimaging, National Research Council, Naples, Italy

**Keywords:** multiple sclerosis, progressive multifocal leukoencephalopathy, neuroinflammation, neuroimmunology, MRI, susceptibility-weighted imaging

## Abstract

**Background:**

Brain MRI plays an essential role in both diagnosis and follow-up of the JC virus infection of the brain. Recently, MR studies with susceptibility-weighted imaging (SWI) sequences have shown hypointensities in U-fibers adjacent to white matter (WM) lesions of progressive multifocal leukoencephalopathy (PML). This finding has been confirmed with the use of quantitative susceptibility mapping (QSM), allowing to hypothesize a paramagnetic effect in these regions. Here, we report the first longitudinal assessment of QSM and R2* maps in natalizumab-associated PML to evaluate serial changes in susceptibility contrast images and their role in PML diagnosis and follow-up.

**Case presentation:**

We report the case of a 42-year-old woman with multiple sclerosis (MS) who eventually developed, after the 28th natalizumab infusion, subacute cognitive decline and received a laboratory-confirmed diagnosis of PML, leading to immediate drug discontinuation. Three months later, she suffered a new clinical exacerbation, with a brain scan revealing significant inflammatory activity compatible with the radiological diagnosis of an Immune Reconstitution Inflammatory Syndrome (IRIS). She was then treated with corticosteroids until the clinico-radiological spectrum became stable, with the final outcome of a severe functional impairment. Quantitative maps obtained in the early symptomatic stage clearly showed increased QSM and R2* values in the juxtacortical WM adjacent to PML lesions, which persisted during the subsequent disease course.

**Discussion and conclusion:**

High QSM and R2* values in U-fibers adjacent to WM lesions were early and seemingly time-independent radiological findings in the presented PML case. This, coupled to the known absence of significant paramagnetic effect of new active MS lesions, could support the use of quantitative MRI as an additional tool in the diagnosis and follow-up of natalizumab-related PML in MS.

## Background

Progressive multifocal leukoencephalopathy (PML) is a rare and severe demyelinating disease of the central nervous system caused by the reactivation of JC virus (JCV) in immunosuppressed patients (1).

In recent years, PML has gained increasing attention due to its association with natalizumab treatment in multiple sclerosis (MS) (2–4) and its prognosis, once regarded as invariably poor, seemingly ameliorates with early diagnosis and adequate treatment (5).

Neuroimaging plays a pivotal role in both diagnosis and follow-up of JCV infection and brain MRI has been included in PML diagnostic workflow, due to its high-sensitivity in the screening of suspected patients, even in a presymptomatic stage (6, 7).

Conventional MRI findings of PML are relatively well-known (8–11). Recently, some authors investigated brain magnetic susceptibility changes in PML, proposing the use of susceptibility contrast [namely, susceptibility-weighted imaging (SWI)] as an additional diagnostic tool (12–14). However, to date, no data are available on the quantitative longitudinal assessment of susceptibility changes in PML and their possible use as a biomarker of disease progression.

In contrast to SWI, quantitative susceptibility mapping (QSM) and R2* mapping offer the possibility of a quantitative, and therefore more consistent, evaluation of brain susceptibility changes. Here, we describe the first longitudinal use of QSM and relaxometry maps in a patient with MS who developed a natalizumab-related PML.

## Case Presentation

We report the case of a 42-year-old woman with a 10-year history of relapsing remitting-MS, treated with natalizumab in the last 2 years. She was JCV antibody-positive and had previously received another disease modifying therapy (interferon beta-1a/Rebif 44).

After the 28th infusion of natalizumab, she eventually developed subacute cognitive decline, confirmed by neuropsychological tests. Approximately 2 weeks after this clinical onset, a brain MRI scan revealed the presence of multiple new lesions, highly suggestive of PML. Natalizumab was immediately discontinued, she was hospitalized and JCV DNA copies were found in cerebrospinal fluid (2,322 copies/mL), leading to a definite diagnosis of PML (6).

Three months later, prompted by further cognitive deterioration and subacute right hemiplegia, another brain MRI scan revealed significant inflammatory activity, compatible with the clinico-radiological diagnosis of an Immune Reconstitution Inflammatory Syndrome (IRIS).

She subsequently received corticosteroids (several 3–5 days cycles of 1 g/die i.v. bolus of methylprednisolone, alternating with 25 mg/die of prednisone *per os*), until the clinico-radiological spectrum became stable, and she was moved to a neuro-rehabilitation center.

The final clinical outcome was a severe functional impairment, with impossibility of independent ambulation, right hemiplegia, incoordination, mixed aphasia, cognitive decline, and an overall Expanded Disability Status Scale score of 7.0, compared with the baseline value of 2.5.

Since the clinical onset, the patient was closely followed-up in our center with several brain MRI scans, three of them with contrast administration, respectively at 35, 91, and 280 days from the first diagnostic examination.

Written informed consent was obtained from the patient for the publication of this case report.

MR data were collected on a 3-T scanner (Trio, Siemens Medical Systems, Erlangen, Germany).

In all scans, along with the routine clinical sequences, an unenhanced 3D double-echo FLASH sequence (TR = 28 ms; TE_1_ = 7.63 ms; TE_2_ = 22.14 ms; voxel size = 0.65 mm × 0.65 mm × 1.3 mm; 128 axial slices) was acquired with a flip angle of 20° to provide QSM and R2* maps, as already described in detail (15–17).

**Figure 1:**
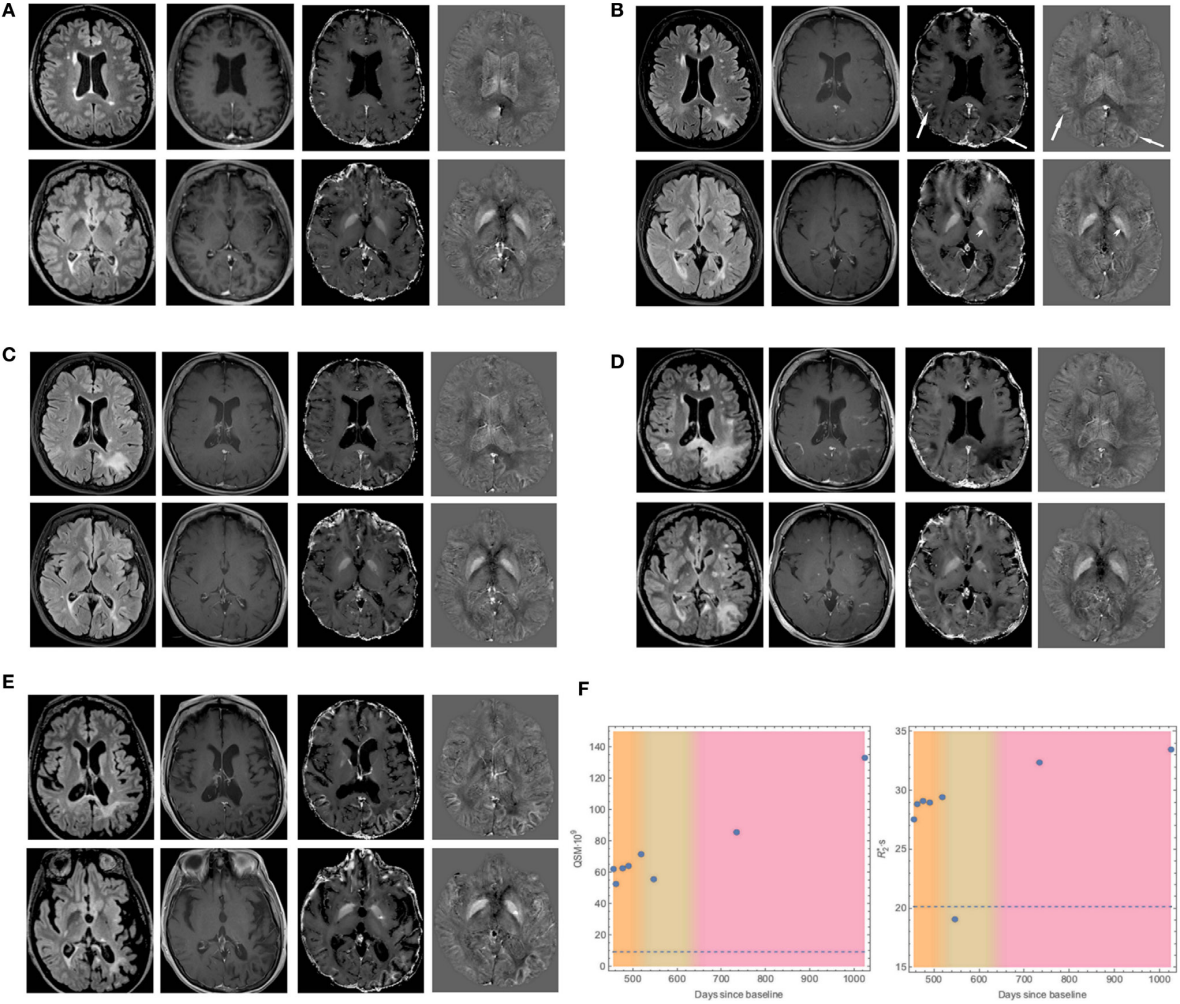
Evolution of MRI findings in our case of natalizumab-related progressive multifocal leukoencephalopathy (PML). Axial fluid-attenuated inversion recovery (FLAIR, first column), contrast-enhanced T1-weighted images (second column), R2* maps (third column), and quantitative susceptibility maps (QSM, fourth column), of two levels (ventricular bodies and basal ganglia planes) from brain MRI scans performed in a phase of multiple sclerosis stability **(A)**, in the early symptomatic PML phase **(B)**, at a PML follow-up **(C)**, when Immune Reconstitution Inflammatory Syndrome (IRIS)-PML ensued **(D)**, and at a chronic stage **(E)**. **(A)** Multiple periventricular and juxtacortical FLAIR-hyperintense demyelinating lesions, non-enhancing after i.v. gadolinium administration, and without significant susceptibility modifications on R2* or QSM maps. **(B)** Evidence of new FLAIR-hyperintense bilateral parieto-occipital subcortical lesions, with mild contrast enhancement and clearly increased values of R2* and QSM in the adjacent U-fibers (arrows). Furthermore, a small non-enhancing, FLAIR-hyperintense lesion in the left globus pallidus is present, showing mildly decreased values on R2* and QSM maps (arrowheads). **(C)** Progressive extension of the previously described lesions showing lack of significant contrast-enhancement and constantly high R2* and QSM values in adjacent U-fibers. The left pallidal lesion now shows a mild increase in susceptibility on R2* and QSM maps. **(D)** Further extension of FLAIR-hyperintensities with most of the lesions vividly enhancing after contrast administration. U-fibers high values on R2* and QSM maps are still present, whereas the susceptibility changes in the left globus pallidus become more evident. **(E)** Reduction of the lesion load with significantly increased values on R2* and QSM maps of adjacent U-fibers, associated with brain atrophy. The left globus pallidus’ lesion shows a punctate hypointense signal on FLAIR images, corresponding to a significant increase of R2* and QSM values. **(F)** Plots showing the time evolution of QSM and R2* values in the U-fibers adjacent to the left parieto-occipital subcortical lesion, with reference to the baseline (dotted line). The three colored bands represent different phases of the disease (PML, PML–IRIS, and chronic stage).

A complete depiction of the evolution of MRI findings during the disease course is shown in Figure 1, where four MRI scans are displayed, in comparison with a pre-PML baseline examination, performed after 14 natalizumab infusions, while the patient was asymptomatic (Figure 1A).

In summary, the first brain MRI performed shortly after clinical onset revealed new bilateral parieto-occipital subcortical lesions, with mild contrast-enhancement and clearly increased susceptibility and R2* values in the adjacent U-fibers. Moreover, a new fluid-attenuated inversion recovery (FLAIR)-hyperintense lesion was present in the left globus pallidus, with no significant paramagnetic effect (Figure 1B).

Despite natalizumab discontinuation, a follow-up MRI scan, performed 35 days later, revealed progressive extension of the subcortical white matter (WM) lesions with persistently high QSM and R2* values in the adjacent U-fibers. Besides, the left pallidal lesion showed a mild increase of both susceptibility and R2*, reflecting a more marked paramagnetic effect (Figure 1C).

Following a clinical exacerbation, another brain MRI scan performed 91 days after the first diagnostic examination showed further increase in the multifocal extension, with changes in the contrast-enhancement pattern, suggestive of intense inflammatory activity due to IRIS. QSM anomalies of U-fibers adjacent to WM lesions were still present, even if more blurred, while susceptibility changes in the left globus pallidus became more evident (Figure 1D).

Finally, in a chronic stage, 280 days after the first examination, a follow-up brain MRI (Figure 1E) revealed partial regression of the lesions, associated to a moderate parieto-occipital atrophy, and even higher QSM and R2* values in the affected U-fibers. Furthermore, the left pallidal lesion showed a punctate hypointense focus on FLAIR images, corresponding to a significant increase in susceptibility on QSM and R2* maps.

Plots showing the time evolution of QSM and R2* values in the U-fibers adjacent to the left parieto-occipital subcortical lesion are shown in Figure 1F.

## Discussion

Brain MRI is essential for the diagnosis and monitoring of PML, and several conventional MRI features have been reported as characteristics of this condition, including multifocal WM lesions involving U-fibers, punctate and/or rim-like enhancement after gadolinium injection, and minimal/absent mass effect (8, 9).

Furthermore, some non-conventional MRI techniques (e.g., MR-spectroscopy and ultra-high field MRI) have been proposed as an additional diagnostic tool in this condition (18–20), and the role of susceptibility contrast imaging has been recently investigated.

In particular, previous studies have reported the presence, on SWI sequences, of hypointensities in U-fibers adjacent to the WM lesions of PML (13, 14). This finding has been confirmed by the observation of clearly increased values in U-fibers on QSM maps, allowing to hypothesize a paramagnetic effect in the juxtacortical regions adjacent to PML lesions (12). However, these findings have only been reported in cross-sectional reports, evaluating imaging findings at a specific time point.

To the best of our knowledge, no studies have been performed using a quantitative MRI method at different time points, covering the entire history of the disease to evaluate possible susceptibility changes that may help in the diagnosis or in monitoring the clinical evolution of these patients.

In our case, increased QSM values in U-fibers adjacent to WM lesions, suggesting a paramagnetic effect, were a constant finding. This feature was independent from the clinical course of the disease, being already present in the early phase, when the appearance of new lesions could mimic MS recurrence rather than the occurrence of a PML, and persisting in chronic stages. This finding, coupled with the known absence of significant paramagnetic effect of new active MS lesion (21, 22), allows us to encourage the inclusion of susceptibility contrast imaging, and in particular of QSM, in clinical routine examination, since the presence of a clear paramagnetic effect could further help in differentiating PML lesions from MS progression.

The meaning of these susceptibility changes has not yet been fully understood. Indeed, high amounts of iron have been reported within oligodendrocytes and myelin sheaths at the cortical–subcortical junction, in both healthy and MS patients’ brains (23). In MS plaques, iron is released by oligodendrocytes and myelin destruction and, in the chronic course of the plaque, it accumulates within activated macrophages and microglia, with an increasing paramagnetic effect (21, 22). Our findings support the hypothesis that a similar mechanism of iron accumulation could occur in PML, in which however a different inflammatory pattern, characterized by more pronounced myelin degeneration, could lead to an increase in intracellular iron deposition with stronger and earlier detectable susceptibility changes (14). During the PML–IRIS phase, a slight descent of QSM values (which however were still markedly higher than baseline), was most likely due to macrophages dilution in the abundant inflammatory edema (as suggested by the conspicuous R2* drop).

In addition, we reported the presence of a lesion in the left globus pallidus, characterized by slowly progressive increase of QSM and R2* values. Previous studies have described susceptibility changes in basal ganglia of PML patients, characterized mainly by a diffuse, although asymmetrical, low signal on SWI typically attributed to iron deposition (13, 14). In our case, no significant global susceptibility changes were present in deep gray matter nuclei. These apparently conflicting results may be related to the absence, in our patient, of an extensive involvement of the deep WM adjacent to the basal ganglia. Instead, susceptibility changes in the left globus pallidus are more likely attributable to an intralesional microhemorrage, also reported as a rare finding in PML (1, 24), rather than to a focal iron accumulation within the lesion.

Finally, some limitations should be considered in the present report. In particular, histopathological confirmation is lacking in our case, which would have allowed to directly verify our hypotheses. Furthermore, this is a single-subject evaluation, although studied at several time points, with all the associated limits. For this reason, future longitudinal studies including a group of PML patients are warranted, to further corroborate our findings.

## Conclusion

We support the hypothesis that susceptibility contrast imaging could represent a valid aid in the diagnosis of natalizumab-related PML in MS, even at an early stage, and therefore should be included in the MRI evaluation of patients with such clinical suspicion, or even in the routine imaging surveillance in this population.

## Ethics Statement

This study was carried out in accordance with the recommendations of “name of guidelines, name of committee” with written informed consent from the subject. The subject gave written informed consent in accordance with the Declaration of Helsinki. Since this was an observational case study, a formal review by the local Institutional Review Board was not required.

## Author Contributions

GPontillo and SC: study concept and design; analysis and interpretation. RL, AR, and VM: acquisition of data. PB: analysis and interpretation. ET: critical revision; study supervision. GPalma: analysis and interpretation; critical revision; and study supervision.

## Conflict of Interest Statement

The authors declare that the research was conducted in the absence of any commercial or financial relationships that could be construed as a potential conflict of interest.
